# Adaptive Federated IMM Filter for AUV Integrated Navigation Systems

**DOI:** 10.3390/s20236806

**Published:** 2020-11-28

**Authors:** Weiwei Lyu, Xianghong Cheng, Jinling Wang

**Affiliations:** 1 School of Instrument Science & Engineering, Southeast University, Nanjing 210096, China; lvww0220@seu.edu.cn; 2 Key Laboratory of Micro-Inertial Instrument and Advanced Navigation Technology, Ministry of Education, Southeast University, Nanjing 210096, China; 3 School of Civil and Environmental Engineering, University of New South Wales, Sydney, NSW 2052, Australia; jinling.wang@unsw.edu.au

**Keywords:** AUV, federated Kalman filter, integrated navigation, information sharing coefficient, interacting multiple model (IMM)

## Abstract

High accuracy and reliable navigation in the underwater environment is very critical for the operations of autonomous underwater vehicles (AUVs). This paper proposes an adaptive federated interacting multiple model (IMM) filter, which combines adaptive federated filter and IMM algorithm for AUV in complex underwater environments. Based on the performance of each local system, the information sharing coefficient of the adaptive federated IMM filter is adaptively determined. Meanwhile, the adaptive federated IMM filter designs different models for each local system. When the external disturbances change, the model of each local system can switch in real-time. Furthermore, an AUV integrated navigation system model is constructed, which includes the dynamic model of the system error and the measurement models of strapdown inertial navigation system/Doppler velocity log (SINS/DVL) and SINS/terrain aided navigation (SINS/TAN). The integrated navigation experiments demonstrate that the proposed filter can dramatically improve the accuracy and reliability of the integrated navigation system. Additionally, it has obvious advantages compared with the federated Kalman filter and the adaptive federated Kalman filter.

## 1. Introduction

Autonomous underwater vehicle (AUV) is an efficient underwater working platform that has been widely used for various underwater tasks in the areas of oil and gas industry, ocean mapping, archaeological exploration, military reconnaissance missions, search and rescue operations, etc. [1,2,3,4,5]. Over the past few decades, AUV has been developed rapidly, due to its great value of application [4,5]. Accurate navigation and positioning is not only a prerequisite for AUV to perform underwater operations, but also a technical guarantee for its safe return [3,6]. Navigation and positioning is one of the benchmark technologies to evaluate the level of development and the maturity of engineering application of AUV [1,3,4]. However, because of the complexity of ocean environment, how to make AUV reach the operation site accurately and return safely is still a challenging issue [4,5].

Currently, most of AUVs adopt a strapdown inertial navigation system (SINS) as the reference navigation system [3,7]. SINS is an independent navigation system that is able to provide comprehensive navigation information, including the velocity, position, and attitude [8,9]. However, because of the errors of inertial sensors, the navigation solution of SINS diverges over time [10,11]. Hence, SINS is often aided by other navigation systems, such as Doppler velocity log (DVL) [2,7,12], magnetometer [7], depth sensor [11], terrain aided navigation (TAN) [13,14,15,16], acoustic long baseline (LBL) [17], vision navigation [18], and so on [6]. In [11], an underwater navigation system composed of a high-rate SINS and low-rate aided sensors was designed, and the aided sensors consisted of a DVL, an inclinometer, a depth sensor, and a global positioning system (GPS). In [7], the Technion autonomous underwater vehicle (TAUV) was discussed, and its navigation system was composed of a SINS integrated by a DVL, a pressure sensor, and a magnetometer. Due to the lack of underwater maps for terrain based navigation, Nygren et al. [15] proposed a new method that uses a multibeam sonar and a linear Kalman filter to accommodate low sampling frequency. The above studies utilize various navigation sensors to aid SINS, and the navigation performance of AUVs has been effectively improved. 

The decentralized filtering methods in a multi-sensor information fusion system have attracted the increasing attention of researchers [19]. Compared with the centralized filtering methods, the decentralized filtering methods dramatically reduce the computational effort and increase the fault-tolerant capability of the integrated navigation system [20,21]. Among the decentralized filtering methods, the decentralized federated filter created by Carlson [20,21,22,23] is most well known.

According to the principle of the decentralized federated filter, the local filters are designed to be suboptimal filters that provide the global optimal estimation [20,24]. In [21], Carlson discussed a federated filter that was applied to integrated and fault-tolerant navigation systems. The superiority of fault tolerance, estimation accuracy, and computation speed was demonstrated by the numerical simulation results and real-time implementations. However, the information distribution principle in the federated filter was designed to be at a fixed ratio, which means that each local system has a fixed information coefficient [19,25,26]. However, in practical applications, the performance and estimated accuracy of the local system is continually changing with the complex navigation environment [3,7,27,28]. In order to increase the performance of the federated filter, Shen et al. [25] proposed a new adaptive federated Kalman filter with time-varying information sharing coefficients on the basis of observability analysis for unmanned ground vehicles (UGV) integrated navigation. Xiong et al. [19] designed a novel dynamic vector-form information sharing method based on the analysis of the error covariance matrix and the observability matrix for the federated filter in a highly dynamic environment. Wang et al. [29] proposed an adaptive information sharing factor federated filter (AISFF) which can adaptively adjust the information sharing factor to improve the reliability of autonomous navigation for Unmanned Surface Vehicles (USVs). The above research has optimized the information distribution principle of federated filter, and the navigation accuracy has been effectively improved. However, when underwater environment in concerned, ocean currents, turbulence, changes in salinity and temperature, and other underwater phenomena can influence the models of AUV integrated navigation systems [1,4,30]. In fact, as the errors and disturbances are usually time-varying and change with the underwater environment, it is difficult to model them accurately [5,7,31], which will lead to greater inaccuracies of AUV integrated navigation systems. Therefore, how to achieve accurate and reliable navigation capability in the underwater environment is a challenging problem to be settled.

In this paper, a new adaptive federated IMM filter is designed for AUV integrated navigation systems. The information sharing coefficient of the adaptive federated IMM filter is dynamically adjusted according to the performance of each local system. Aiming to enhance the accuracy and reliability of AUV operations in a complex underwater environment, this paper studies the IMM algorithm and combines it with the adaptive federated filter. Then, an AUV integrated navigation system model that includes the system error dynamics model, SINS/DVL and SINS/TAN measurement models is constructed. The vehicle integrated navigation experiments demonstrate the high accuracy and reliability of the proposed adaptive federated IMM filtering method.

The structure of this paper is organized as follows: the federated Kalman filter is presented in Section 2. In Section 3, the adaptive federated Kalman filter is designed, then the adaptive federated IMM filter is proposed and analyzed in detail. In Section 4, the AUV integrated navigation system model is constructed in detail. Thirty groups of vehicle integrated navigation experiments are conducted to demonstrate the validity of the proposed method in Section 5. Finally, the conclusions are drawn in Section 6.

## 2. Federated Kalman Filter

The state equation of a linear discrete-time system *i* can be presented as follows [22,32]:

(1) $$X_{k}^{i}=\Phi_{k,k-1}^{i}X_{k-1}^{i}+\Gamma_{k-1}^{i}W_{k-1}^{i}$$

(2) $$Z_{k}^{i}=H_{k}^{i}X_{k}^{i}+V_{k}^{i}$$

where $\Phi_{k,k-1}^{i}$ is the (*n* × *n*) state transition matrix; $\Gamma_{k-1}^{i}$ is the (*n* × *r*) system noise matrix; $W_{k-1}^{i}$ is the (*r* × *r*) system noise matrix; $X_{k}^{i}$ is the (*n* × 1) state estimate; $H_{k}^{i}$ is the (*m* × *n*) measurement matrix; $V_{k}^{i}$ is the measurement noise matrix; $Z_{k}^{i}$ is the (*m* × 1) measurement value.

In Equation (1), both $W_{k-1}^{i}$ and $V_{k}^{i}$ are assumed as the zero-mean Gaussian white noise, and their covariances are:

(3) $$\begin{array}{l} E[W_{j}^{i}W_{k}^{i}{}^{T}]=\sigma_{jk}Q_{k-1}^{i}\\ E[V_{j}^{i}V_{k}^{i}{}^{T}]=\sigma_{jk}R_{k}^{i}\\ E[W_{j}^{i}V_{k}^{i}{}^{T}]=0 \end{array}$$

In Equation (3), $Q_{k-1}^{i}\geq 0$, $R_{k}^{i}>0$, $\sigma_{jk}$ is the Kronecker function, and $\sigma_{jk}=\left\{ \begin{array}{l} 0\;(k\neq j)\\ 1\;(k=j) \end{array}\right.$.

**Figure 1:**
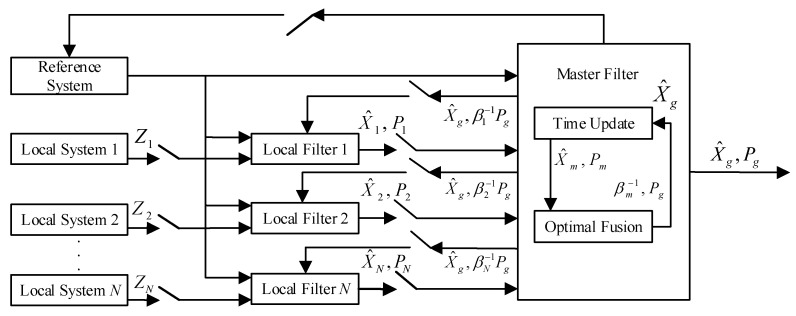
The schematic diagram of the federated Kalman filter.

The federated Kalman filter contains a composite master filter and *N* independent local filters. The schematic diagram of the federated Kalman filter is presented in Figure 1. In summary, the implementation steps of the federated Kalman filter can be presented as follows:

(1) Information sharing:

Define the symbols with the superscript “*g*” as the parameters of the global filter. The system information $Q_{k}^{g}$, $P_{k}^{g}$ and ${\hat{X}}_{k}^{g}$ are allocated according to the following information allocation principles [21,22]:

(4) $$\left\{ \begin{array}{l} Q_{k}^{i}=\beta_{i}^{-1}Q_{k}^{g}\\ P_{k}^{i}=\beta_{i}^{-1}P_{k}^{g}\\ {\hat{X}}_{k}^{i}={\hat{X}}_{k}^{g},i=1\cdots N,m \end{array}\right.$$

where *β_i_* represents the information sharing coefficient, $\beta_{i}>0$. Meanwhile, it satisfies the following information conservation principle:

(5) $$\sum_{i=1}^{N}\beta_{i}+\beta_{m}=1$$

(2) Time updating:

The *N* independent local filters and the master filter and conduct the time updating of the information separately according to the following equations:

(6) $$\left\{ \begin{array}{l} {\hat{X}}_{k/k-1}^{i}=\Phi_{k/k-1}^{i}{\hat{X}}_{k-1}^{i}\\ P_{k/k-1}^{i}=\Phi_{k/k-1}^{i}P_{k-1}^{i}\Phi_{k/k-1}^{i}{}^{T}+\Gamma_{k-1}^{i}Q_{k-1}^{i}\Gamma_{k-1}^{i}{}^{T},i=1\cdots N,m \end{array}\right.$$

(3) Measurement updating:

Because the master filter only plays the role of information fusion and it has no measurement information, the master filter has no process of measurement updating. The *N* independent local filters conduct the measurement updating of the information separately according to the following equations [22,23]:

(7) $$\left\{ \begin{array}{l} {\left(P_{k}^{i}\right)}^{-1}={\left(P_{k/k-1}^{i}\right)}^{-1}+{\left(H_{k}^{i}\right)}^{T}{\left(R_{k}^{i}\right)}^{-1}H_{k}^{i}\\ {\left(P_{k}^{i}\right)}^{-1}{\hat{X}}_{k}^{i}={\left(P_{k/k-1}^{i}\right)}^{-1}{\hat{X}}_{k/k-1}^{i}+{\left(H_{k}^{i}\right)}^{T}{\left(R_{k}^{i}\right)}^{-1}Z_{k}^{i},i=1,2,\cdots ,N \end{array}\right.$$

(4) Information fusion:

The local estimation state of each local filter is fused based on the following two equations, and the global optimal estimation can be obtained:

(8) $$P_{k}^{g}={\left[{\left(P_{k}^{1}\right)}^{-1}+{\left(P_{k}^{2}\right)}^{-1}+\cdots +{\left(P_{k}^{N}\right)}^{-1}+{\left(P_{k}^{m}\right)}^{-1}\right]}^{-1}$$

(9) $$\begin{array}{ll} {\hat{X}}_{k}^{g}= & P_{k}^{g}[{\left(P_{k}^{1}\right)}^{-1}{\hat{X}}_{k}^{1}+{\left(P_{k}^{2}\right)}^{-1}{\hat{X}}_{k}^{2}+\cdots +{\left(P_{k}^{N}\right)}^{-1}{\hat{X}}_{k}^{N}\\ & +{\left(P_{k}^{m}\right)}^{-1}{\hat{X}}_{k}^{m}] \end{array}$$

## 3. Adaptive Federated IMM Filter

### 3.1. Adaptive Federated Kalman Filter

When given a matrix $P_{i}\in C^{m\times n}$, there are unitary matrix $\Upsilon_{i}=\left[\lambda_{i,1}\;\lambda_{i,2}\;\cdots \;\lambda_{i,m}\right]$ and unitary matrix $\Lambda_{i}=\left[\mu_{i,1}\;\mu_{i,2}\;\cdots \;\mu_{i,n}\right]$ that satisfy the following relationship:

(10) $$\Upsilon_{i}{}^{T}P_{i}\Lambda_{i}=\left[\begin{array}{cc} \xi_{i} & 0_{c,n-c}\\ 0_{m-c,c} & 0_{m-c,n-c} \end{array}\right]$$

where $\xi_{i}=diag(\xi_{i,\;1},\xi_{i,\;2},\cdots ,\xi_{i,\;r})$, $\xi_{i,\;1}>\xi_{i,\;2}>\cdots >\xi_{i,\;r}>0$, $c=rank(P_{i})$. The Equation (10) is the singular value decomposition of the matrix $P_{i}$, and $\xi_{i,\;1},\xi_{i,\;2},\cdots ,\xi_{i,\;r}$ are the singular values of the matrix $P_{i}$.

Define $P_{i}({\left(p_{\lambda \mu }\right)}_{n\times n})$ as the covariance matrix of local filter *i*, and $p_{\lambda \mu }$ represents the cross-covariance of local filter *i* between state *λ* and state *μ.* Then the singular value $\xi_{i}$ of the matrix $P_{i}$ contains both the information of auto-covariance and the information of cross-covariance in each system’s state estimation. Therefore, the covariance matrix of local filter *i* contains the information of the estimation error, and it reflects the filtering performance of local filter *i*. Based on the above analysis, this paper determines the information sharing coefficient dynamically from the global filter to the local filter. In local filter *i*, $\xi_{i}(k)$ is defined as the sum of singular values of the covariance matrix $P_{i}(k)$ at filtering step *k*, and it can be expressed as follows:

(11) $$\xi_{i}(k)=\sum_{l=1}^{n}\xi_{i,l}(k)$$

Firstly, the information sharing coefficient $\beta_{m}$ of the master filter is selected, then the information sharing coefficients of local filters can be obtained according to the following equation:

(12) $$\beta_{i}(k)=\frac{1/\xi_{i}(k)}{1/\xi_{1}(k)+1/\xi_{2}(k)+\cdots +1/\xi_{N}(k)}\;(1-\beta_{m})\;,\;i=1,2,\cdots ,N$$

where $\beta_{i}(k)$ represents the information sharing coefficient of local filter *i* at filtering step *k*. 

The information sharing coefficients of the adaptive federated Kalman filter satisfy the following relationship:

(13) $$0\leq \beta_{i}(k)\leq 1,\;\sum_{i=1}^{N}\beta_{i}(k)+\;\beta_{m}=1$$

Consequently, the process of system information distribution can be expressed as:

(14) $$\left\{ \begin{array}{l} Q_{k}^{i}=\beta_{i}{\left(k\right)}^{-1}Q_{k}^{g}\\ P_{k}^{i}=\beta_{i}{\left(k\right)}^{-1}P_{k}^{g}\\ {\hat{X}}_{k}^{i}={\hat{X}}_{k}^{g},i=1\cdots N,m \end{array}\right.$$

### 3.2. Adaptive Federated IMM Filter

When the system has discrete uncertainties together with continuous uncertainties in the dynamic or measurement model, the IMM algorithm is a very effective method [32,33,34,35,36]. The IMM algorithm has shown superior performance with a low computational burden in a variety of applications, such as target tracking [37,38,39,40], mobile node localization [41], and motion planning [42]. However, there are few studies about the application of the IMM algorithm to the underwater navigation system. There are also few studies about the combination of the IMM algorithm and the federated filter. Focused on the above problems, this paper designs a novel adaptive federated IMM filter that combines the adaptive federated filter and the IMM algorithm to enhance the accuracy and reliability of the AUV operations in complex underwater environments. In the proposed method, each local system includes different models, and when the underwater environment changes the model for each local system can switch in real-time. Therefore, the proposed method can use the most accurate mixed model to describe the current state of the local system. 

Assume there are *S* states of motion in the system, then there should be *S* motion models accordingly. The state equation of model *q* can be described as follows:

(15) $$X_{q}(k)=\Phi_{q}(k|k-1)X_{q}(k-1)+\Gamma_{q}(k-1)W_{q}(k-1)$$

In addition, the measurement equation of model *q* can be presented as follows:

(16) $$Z_{q}(k)=H_{q}(k)X_{q}(k)+V_{q}(k)$$

where $W_{q}(k-1)$ is the uncorrelated zero-mean Gaussian white noise, and its conversance matrix is $Q_{q}(k-1)$. The Markov probability transfer matrix determines the transfer between each of the model, and the element $e_{pq}$ represents the probability of the model *p* transferring to the model *q*. The probability transfer matrix is represented as follows:

(17) $$E=\left[\begin{array}{ccc} e_{11} & \cdots & e_{1s}\\ \cdots & \cdots & \cdots \\ e_{s1} & \cdots & e_{ss} \end{array}\right]$$

The IMM algorithm runs recursively, and each recursion is mainly divided into the following four steps:

(1) Interactive input (model *q*):

Based on the estimation state ${\hat{X}}_{p}(k-1)$ and the model probability $\mu_{p}(k-1)$ of each filter at the filtering step *k* − 1, the mixed estimation state ${\hat{X}}_{0q}(k-1)$, and the covariance matrix $P_{0q}(k-1)$ is obtained, and the mixed estimation results are regarded as the initial states of the current recursion. The specific parameters are calculated as follows:

The prediction probability of the model *q* can be represented as

(18) $${\bar{c}}_{q}=\sum_{p=1}^{s}e_{pq}\mu_{p}(k-1)$$

The mixed probability of the model *p* transfer to the model *q* is

(19) $$\mu_{pq}(k-1)=e_{pq}\mu_{p}(k-1)/{\bar{c}}_{q}$$

The mixed estimation state of the model *q* is represented as

(20) $${\hat{X}}_{0q}(k-1)=\sum_{p=1}^{s}{\hat{X}}_{p}(k-1)\mu_{pq}(k-1)$$

Then the mixed covariance matrix estimation of the model *q* is represented as

(21) $$\begin{array}{ll} P_{0q}(k-1)= & \sum_{p=1}^{s}\mu_{pq}(k-1)\{P_{p}{\left(k-1\right)}^{{}^{{}^{}}}+\left[{\hat{X}}_{p}(k-1)-{\hat{X}}_{0q}(k-1)\right]\\ & \cdot {\left[{\hat{X}}_{p}(k-1)-{\hat{X}}_{0q}(k-1)\right]}^{T}\} \end{array}$$

(2) Kalman filtering (model *q*):

Take ${\hat{X}}_{0q}(k-1)$, $P_{0q}(k-1)$ and Z(k) as the input of the Kalman filter, then the estimation state ${\hat{X}}_{q}(k)$ and the estimation covariance matrix $P_{q}(k)$ can be updated.

The prediction state is

(22) $${\hat{X}}_{q}(k|k-1)=\Phi_{q}(k|k-1){\hat{X}}_{0q}(k-1)$$

The prediction covariance matrix is

(23) $$P_{q}(k|k-1)=\Phi_{q}(k|k-1)P_{0q}(k-1)\Phi_{q}{\left(k|k-1\right)}^{T}+\Gamma_{q}(k)Q_{q}(k)\Gamma_{q}{\left(k\right)}^{T}$$

The gain of the Kalman filter is

(24) $$K_{q}(k)=P_{q}(k|k-1)H{\left(k\right)}^{T}{\left[H(k)P_{q}(k|k-1)H{\left(k\right)}^{T}+R(k)\right]}^{-1}$$

The estimation state ${\hat{X}}_{q}(k)$ is

(25) $${\hat{X}}_{q}(k)={\hat{X}}_{q}(k|k-1)+K_{q}(k)\left[Z(k)-H(k)X_{q}(k|k-1)\right]$$

Then the estimation covariance matrix $P_{q}(k)$ is

(26) $$P_{q}(k)=\left[I-K_{q}(k)H(k)\right]P_{q}(k|k-1)$$

(3) Model probability updating (model *q*):

The likelihood function is applied to update the model probability $\mu_{q}(k)$, the likelihood function of the model *q* can be presented as

(27) $$\vartheta_{q}(k)=\frac{1}{{\left(2\pi \right)}^{n/2}{\left|T_{q}(k)\right|}^{1/2}}\exp\left\{-\frac{1}{2}\rho_{q}{\left(k\right)}^{T}T_{q}^{-1}(k)\rho_{q}(k)\right\}$$

where

(28) $$\rho_{q}(k)=Z(k)-H(k){\hat{X}}_{q}(k|k-1)$$

(29) $$T_{q}(k)=H(k)P_{q}(k|k-1)H{\left(k\right)}^{T}+R(k)$$

Then, the probability of the model *q* is

(30) $$\mu_{q}(k)=\vartheta_{q}(k){\bar{c}}_{q}/c$$

where *c* is a normalization constant, and $c=\sum_{q=1}^{s}\vartheta_{q}(k){\bar{c}}_{q}$.

(4) Interactive output:

Based on the probability of each model, the estimation result of each filter can be combined, then the mixed estimation state $\hat{X}(k)$ and the mixed covariance matrix estimation P(k) can be calculated.

Consequently, the mixed estimation state can be presented as

(31) $$\hat{X}(k)=\sum_{q=1}^{s}{\hat{X}}_{q}(k)\mu_{q}(k)$$

In addition, the mixed estimation covariance matrix can be presented as

(32) $$P(k)=\sum_{q=1}^{s}\mu_{q}(k)\left\{P_{q}(k|k-1)+\left[{\hat{X}}_{q}(k)-\hat{X}(k)\right]{\left[{\hat{X}}_{q}(k)-\hat{X}(k)\right]}^{T}\right\}$$

**Figure 2:**
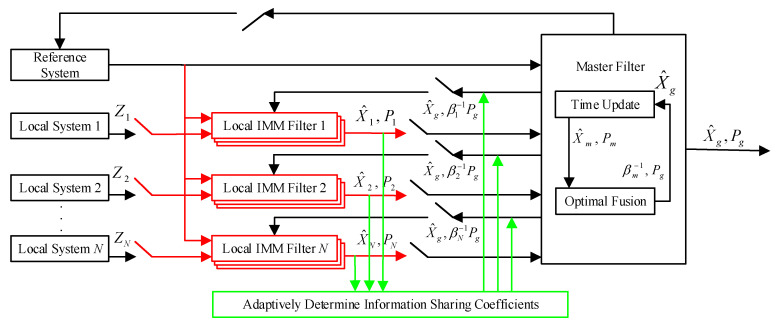
The schematic diagram of the adaptive Federated IMM filter.

In general, the whole output results of the IMM algorithm are the combined values of all filters, and the weight factor of each filter is the model probability that represents the accuracy of the current motion state of the system. The schematic diagram of the proposed adaptive federated IMM filter is presented in Figure 2.

## 4. AUV Integrated Navigation System Model

### 4.1. System Error Dynamics Model

This paper defines “east-north-up (ENU)” as the navigation frame, and “right-forward-up” as the body frame. The SINS’ state equation in a continuous-time system can be constructed as follows:

(33) $$\dot{X}(t)=F(t)X(t)+G(t)W(t)$$

where X(t) is the (*n* × 1) state estimation, $\dot{X}(t)$ is the (*n* × 1) one step predicted state, F(t) is the (*n* × *n*) state transition matrix, G(t) is the system noise matrix, and W(t) is the zero-mean Gaussian white noise.

In the model of the AUV integrated navigation system, fifteen dimensions’ states are selected to establish the equation of states. The system’s states can be expressed as follows:

(34) $$X(t)={\left[\phi_{E},\phi_{N},\phi_{U},\delta V_{E},\delta V_{N},\delta V_{U},\delta L,\delta \lambda ,\delta h,\varepsilon_{x},\varepsilon_{y},\varepsilon_{z},\nabla_{x},\nabla_{y},\nabla_{z}\right]}^{T}$$

where *φ_E_*, *φ_N_*, and *φ_U_* are the misalignment angles; *δV_E_*, *δV_N_* and *δV_U_* are the velocity errors; *δL*, *δλ* and *δh* are the latitude error, longitude error, and height error, respectively; *ε_x_*, *ε_y_*, and *ε_z_* are the gyro drifts; and ∇*_x_*, ∇*_y_* and ∇*_z_* are the accelerometer biases.

The instruction angular velocity and gyro bias influence the attitude angle error of the SINS. The attitude error equations in ENU axes are as follows:

(35) $$\begin{array}{ll} {\dot{\phi }}_{E}= & \phi_{N}(\omega_{ie}\sin L+\frac{V_{E}}{R_{N}+h}\tan L)-\phi_{U}(\omega_{ie}\cos L+\frac{V_{E}}{R_{N}+h})-\frac{\delta V_{N}}{R_{M}+h}\\ & +\delta h\frac{V_{N}}{{\left(R_{M}+h\right)}^{2}}-\varepsilon_{E} \end{array}$$

(36) $$\begin{array}{ll} {\dot{\phi }}_{N}= & -\phi_{E}(\omega_{ie}\sin L+\frac{V_{E}}{R_{N}+h}\tan L)-\phi_{U}\frac{V_{N}}{R_{M}+h}-\delta L\omega_{ie}\sin L+\frac{\delta V_{E}}{R_{N}+h}\\ & \;-\delta h\frac{V_{E}}{{\left(R_{N}+h\right)}^{2}}-\varepsilon_{N} \end{array}$$

(37) $$\begin{array}{ll} {\dot{\phi }}_{U} & =\phi_{E}(\omega_{ie}\cos L+\frac{V_{E}}{R_{N}+h})+\phi_{N}\frac{V_{N}}{R_{M}+h}+\delta L(\omega_{ie}\cos L+\frac{V_{E}}{R_{N}+h}\sec^{2}L)\\ & \;+\frac{\delta V_{E}}{R_{N}+h}\tan L\begin{array}{c} -\delta h\frac{V_{E}\tan L}{{\left(R_{N}+h\right)}^{2}} \end{array}-\varepsilon_{U} \end{array}$$

where *L* is the latitude, *R_M_* is the radius of curvature in meridian, and *R_N_* is the radius of curvature in prime vertical. 

The output of inertial components is processed by the SINS to obtain the navigation data. The analytical relationship between the accelerometer output and the carrier’s velocity can be described by the velocity error equation. The velocity error equations in ENU axes are as follows:

(38) $$\begin{array}{ll} \delta {\dot{V}}_{E}= & \phi_{U}f_{N}-\phi_{N}f_{U}+\delta V_{N}\left(2\omega_{ie}\sin L+\frac{V_{E}\tan L}{R_{N}+h}\right)-\delta V_{U}\left(2\omega_{ie}\cos L+\frac{V_{E}}{R_{N}+h}\right)\\ & \;+\delta V_{E}\frac{V_{N}\tan L-V_{U}}{R_{N}+h}+\delta L\left[2\omega_{ie}\left(V_{U}\sin L+V_{N}\cos L\right)+\frac{V_{N}V_{E}}{R_{N}+h}\sec^{2}L\right]\\ & \;+\delta h\frac{V_{U}V_{E}-V_{N}V_{E}\tan L}{{\left(R_{N}+h\right)}^{2}}+\nabla_{E} \end{array}$$

(39) $$\begin{array}{ll} \delta {\dot{V}}_{N}= & \phi_{E}f_{U}-\phi_{U}f_{E}-\delta V_{N}\frac{V_{U}}{R_{M}+h}-\delta V_{U}\frac{V_{N}}{R_{M}+h}-\delta V_{E}\cdot 2\left(\omega_{ie}\sin L+\frac{V_{E}\tan L}{R_{N}+h}\right)\\ & \;-\delta L\left(2V_{E}\omega_{ie}\cos L+\frac{V_{E}^{2}}{R_{N}+h}\sec^{2}L\right)+\delta h\left[\frac{V_{N}V_{U}}{{\left(R_{M}+h\right)}^{2}}+\frac{V_{E}^{2}\tan L}{{\left(R_{N}+h\right)}^{2}}\right]+\nabla_{N} \end{array}$$

(40) $$\begin{array}{ll} \delta {\dot{V}}_{U}= & -\phi_{E}f_{N}+\phi_{N}f_{E}+\delta V_{N}\frac{2V_{N}}{R_{M}+h}+\delta V_{E}\cdot 2\left(\omega_{ie}\cos L+\frac{V_{E}}{R_{N}+h}\right)\\ & \;-\delta L\cdot 2V_{E}\omega_{ie}\sin L-\delta h\left[\frac{V_{N}^{2}}{{\left(R_{M}+h\right)}^{2}}+\frac{V_{E}^{2}}{{\left(R_{N}+h\right)}^{2}}\right]+\nabla_{U} \end{array}$$

The position error equations in ENU axes are as follows:

(41) $$\delta \dot{L}=\frac{\delta V_{N}}{R_{M}+h}-\delta h\frac{V_{N}}{{\left(R_{M}+h\right)}^{2}}$$

(42) $$\delta \dot{\lambda }=\frac{\delta V_{E}}{R_{N}+h}\sec L+\delta L\frac{V_{E}}{R_{N}+h}\tan L\sec L-\delta h\frac{V_{E}\sec L}{{\left(R_{N}+h\right)}^{2}}$$

(43) $$\delta \dot{h}=\delta V_{U}$$

### 4.2. System Measurement Model

The measurement equation of the AUV integrated navigation system can be expressed as follows:

(44) Z(t)=H(t)X(t)+V(t)

In the AUV integrated navigation system, SINS is chosen as the basic navigation system, and the aided navigation sensor can be chosen according to the actual situation. In this paper, DVL and TAN are chosen as the aided navigation sensors. Therefore, there will be two integrated system measurement equations that are discussed respectively as follows:

(1) SINS/DVL measurement equation

The DVL provides velocity information for the AUV. As shown in Equation (45), the measurement information of the SINS/DVL measurement equation consists of the difference between the east velocity $V_{SE}$, north velocity $V_{SN}$ and up velocity $V_{SU}$ exported by the SINS and the east velocity $V_{DE}$, north velocity $V_{DN}$ and up velocity $V_{DU}$ exported by the DVL.

(45) $$\begin{array}{ll} Z_{SINS/DVL}(t) & =\left[\begin{array}{l} V_{SE}-V_{DE}\\ V_{SN}-V_{DN}\\ V_{SU}-V_{DU} \end{array}\right]=\left[\begin{array}{l} \delta V_{SE}-\delta V_{DE}+\zeta_{E}\\ \delta V_{SN}-\delta V_{DN}+\zeta_{N}\\ \delta V_{SU}-\delta V_{SU}+\zeta_{U} \end{array}\right]\\ & \;=\left[\begin{array}{ccccc} 0_{1\times 3} & 1 & 0 & 0 & 0_{1\times 9}\\ 0_{1\times 3} & 0 & 1 & 0 & 0_{1\times 9}\\ 0_{1\times 3} & 0 & 0 & 1 & 0_{1\times 9} \end{array}\right]X(t)+V_{SINS/DVL} \end{array}$$

where $\delta V_{SE}$, $\delta V_{SN}$, $\delta V_{SU}$ are the east velocity error, north velocity error and up velocity error of the SINS, respectively; $\delta V_{DE}$, $\delta V_{DN}$, $\delta V_{SU}$ are the east velocity error, north velocity error and up velocity error of the DVL, respectively; $\zeta_{E}$, $\zeta_{N}$, $\zeta_{U}$ are the measurement noises of the SINS/DVL integrated navigation system, they are the independent zero-mean Gaussian white-noise sequences.

(2) SINS/TAN measurement equation

The TAN provides position information for the AUV. As shown in Equation (46), the measurement information of the SINS/TAN measurement equation consists of the difference between the latitude $L_{SINS}$, longitude $\lambda_{SINS}$ and height $h_{SINS}$ exported by the SINS and the latitude $L_{TAN}$, longitude $\lambda_{TAN}$ and height $h_{TAN}$ exported by the TAN.

(46) $$\begin{array}{ll} Z_{SINS/TAN}(t) & =\left[\begin{array}{l} L_{SINS}-L_{TAN}\\ \lambda_{SINS}-\lambda_{TAN}\\ h_{SINS}-h_{TAN} \end{array}\right]=\left[\begin{array}{l} \delta L_{SINS}-\delta L_{TAN}+\eta_{L}\\ \delta \lambda_{SINS}-\delta \lambda_{TAN}+\eta_{\lambda }\\ \delta h_{SINS}-\delta h_{TAN}+\eta_{h} \end{array}\right]\\ & \;=\left[\begin{array}{ccccc} 0_{1\times 6} & 1 & 0 & 0 & 0_{1\times 6}\\ 0_{1\times 6} & 0 & 1 & 0 & 0_{1\times 6}\\ 0_{1\times 6} & 0 & 0 & 1 & 0_{1\times 6} \end{array}\right]X(t)+V_{SINS/TAN} \end{array}$$

where $\delta L_{SINS}$, $\delta \lambda_{SINS}$, $\delta h_{SINS}$ are the latitude error, longitude error and height error of the SINS, respectively; $\delta L_{TAN}$, $\delta \lambda_{TAN}$, $\delta h_{TAN}$ are the latitude error, longitude error and height error of the TAN, respectively; $\eta_{L}$, $\eta_{\lambda }$, $\eta_{h}$ are the measurement noises of the SINS/TAN integrated navigation system, they are the independent zero-mean Gaussian white-noise sequences. 

## 5. Experimental Results and Discussions

### 5.1. Experimental Settings

**Figure 3:**
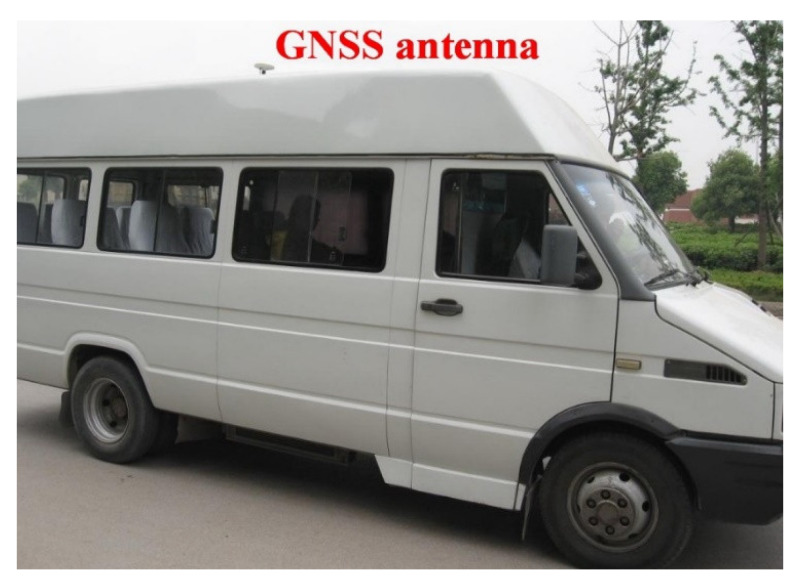
The test vehicle platform.

**Figure 4:**
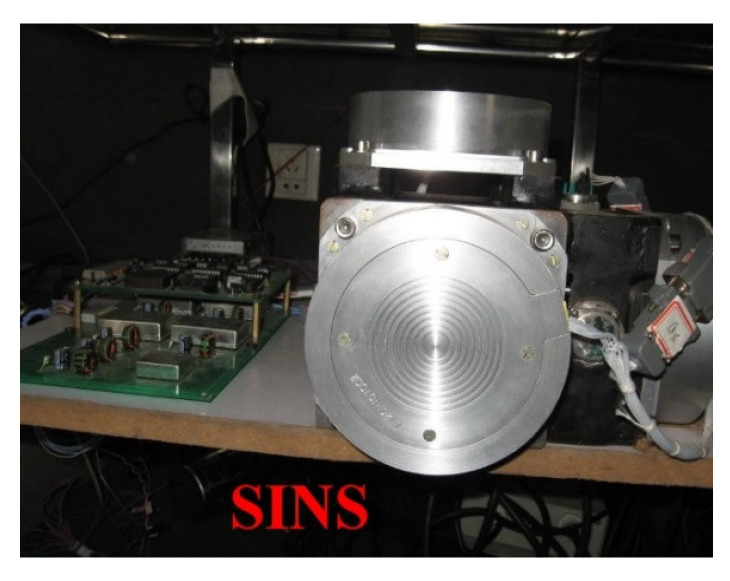
The real picture of the strapdown inertial navigation system (SINS).

**Figure 5:**
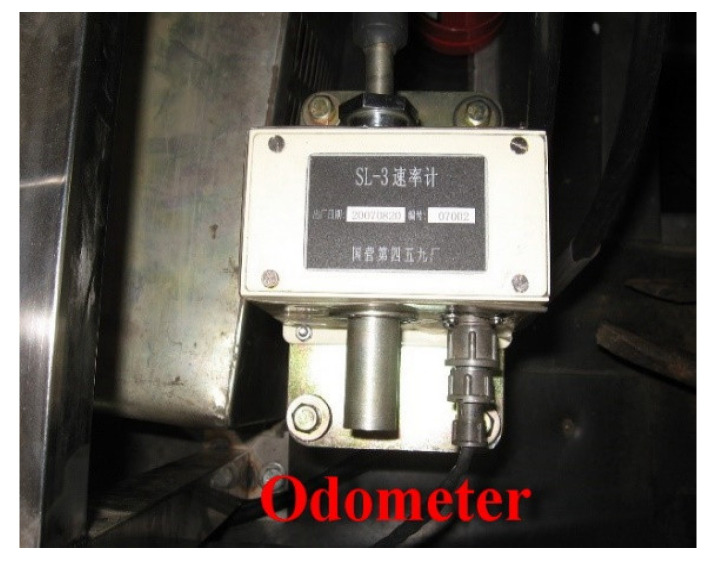
The real picture of the odometer.

In order to verify the proposed adaptive federated IMM filtering method in the AUV integrated navigation system, an integrated navigation experiment in a real system was conducted. The vehicle experiment was carried out outdoors in Beijing, China. The approximate location was east longitude 116° and north latitude 39°. To simulate the output of DVL and the output of TAN in the AUV integrated navigation system, an odometer and a GNSS receiver were adopted. The odometer provided measurement values of velocities and the GNSS receiver provided measurement values of positions, respectively, in the integrated navigation experiment. Figure 3 shows the experimental vehicle platform that includes the SINS, GNSS receiver, odometer, navigation computer, power source, and communication lines. The SINS and the odometer used in the experiments are shown in Figure 4 and Figure 5, respectively.

When the integrated navigation experiment was conducted, the reference baseline information was provided by a high-accuracy GNSS/SINS integrated navigation system consisting of a high-accuracy SINS and a GNSS receiver. The reference information system provides precise information on attitudes, velocities, and positions during the whole experiment. The experimental integrated navigation system that is implemented with the proposed adaptive federated IMM filtering method includes a low-accuracy SINS, a GNSS receiver, and an odometer. The detailed specifications of the instruments in the experimental integrated navigation system are listed in Table 1. The update frequency of the SINS was 200 Hz, and the cycle of attitude solution was 5 ms. The data update cycles of the odometer and the GNSS receiver were all 1 s. To realistically simulate the output of the DVL and the output of the TAN in the underwater environment, noises were added to the outputs of the odometer and the GNSS receiver. To be more specific, during the time intervals of 0–150 s, 150–300 s, and 300–600 s, measurement noises of different levels and different properties were added to the output of the odometer and the output of the GNSS receiver, respectively. Furthermore, the measurement noises added to the output of the odometer and the output of the GNSS receiver were also different from each other. Moreover, the integrated navigation experiment was designed to be close to the experiment in AUV integrated navigation system.

In the adaptive federated IMM filtering method, the specific parameters of the probability transfer matrix ***E*** are set as follows:

(47) $$E=\left[\begin{array}{ccc} 0.9 & 0.05 & 0.05\\ 0.05 & 0.9 & 0.05\\ 0.05 & 0.05 & 0.9 \end{array}\right]$$

The variance matrix of measurement noise in the SINS/DVL integrated navigation system is ***R***_1_ = *diag* [0.1 m/s, 0.1 m/s]^2^. In the proposed adaptive federated IMM filtering method, there are three models for the SINS/DVL integrated navigation system, and the variance matrices of the measurement noise for those three models are ***R***_1_, 3***R***_1_, 8***R***_1_, respectively. 

The variance matrix of measurement noise in the SINS/TAN integrated navigation system is ***R***_2_ = *diag* [10 m, 10 m]^2^. In the proposed adaptive federated IMM filtering method, there are three models for the SINS/TAN integrated navigation system, and the variance matrices of the measurement noise for those three models are ***R***_2_, 5***R***_2_, 10***R***_2_, respectively.

In the integrated navigation experiment, the test vehicle ran on the road, and the power source powered the SINS, the GNSS receiver, the odometer, and the high-accuracy GNSS/SINS reference baseline system. Then the real-time output data of the experimental integrated navigation system and the reference baseline system were transmitted to the navigation computer via communication lines. In the whole integrated navigation experiment, the navigation computer recorded the sensor data in real-time for subsequent processing.

### 5.2. Experimental Results and Discussions

**Figure 6:**
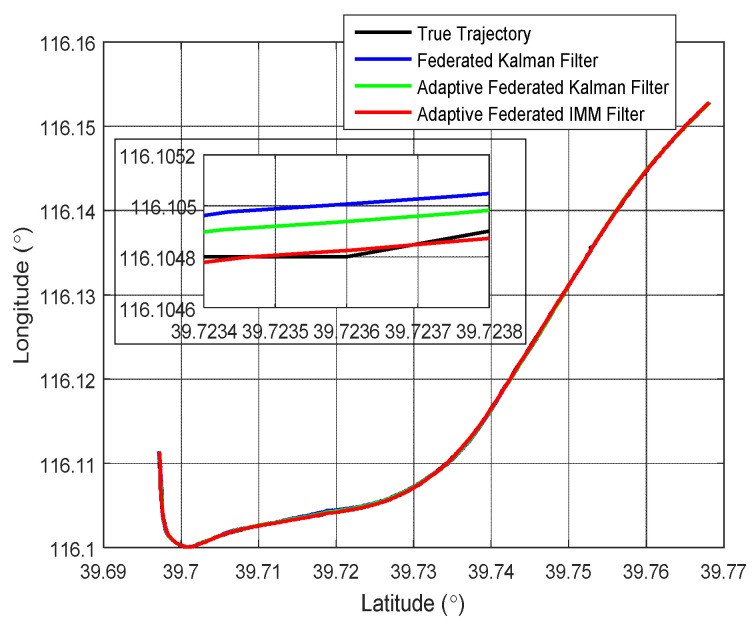
The estimation trajectories of the integrated navigation experiment.

To compare the integrated navigation effect under different filtering methods, the federated Kalman filter, the adaptive federated Kalman filter, and the adaptive federated IMM filter were separately applied in the real experiment. The integrated navigation experiment lasted for 600 s. Figure 6 shows the estimation trajectories of the integrated navigation experiment by using the three filtering methods. In Figure 6, the black line represents the true trajectory, the blue line represents the estimation trajectory of the federated Kalman filter, the green line represents the estimation trajectory of the adaptive federated Kalman filter, and the red line represents the estimation trajectory of the adaptive federated IMM filter. It can be seen from Figure 6 that all three filtering methods can be used for integrated navigation. But from the partially enlarged figure, the estimation trajectory of the adaptive federated IMM filter is the closest to the true trajectory compared with the estimation trajectories of the adaptive federated Kalman filter and the federated Kalman filter. The estimation trajectory of the adaptive federated Kalman filter is closer to the true trajectory compared with that of the federated Kalman filter. It indicates that among the three filtering methods, the proposed adaptive federated IMM filter can achieve the highest accuracy of integrated navigation, and the adaptive federated Kalman filter is second, followed by the federated Kalman filter.

**Figure 7:**
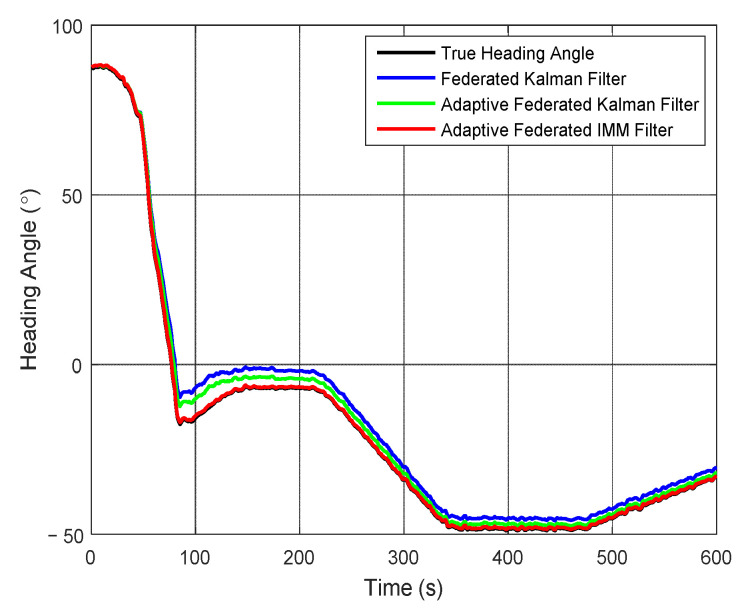
The estimation curves of heading angle.

**Figure 8:**
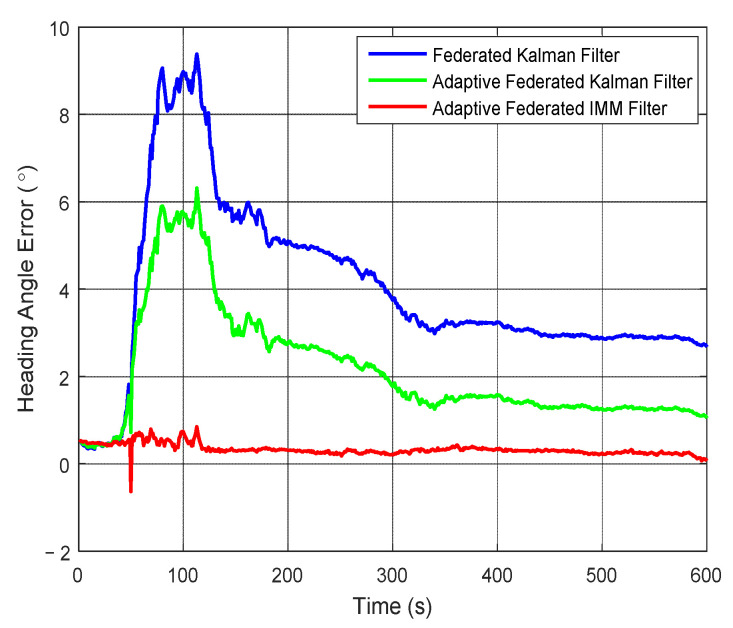
The estimation curves of heading angle error.

To further analyze the results of integrated navigation by using the three filtering methods in detail, the attitude angles, velocities, and positions of the integrated navigation system are presented, respectively, in this paper. The estimation curves of heading angle and heading angle error are presented in Figure 7 and Figure 8, respectively. From Figure 7, the heading angle of the test vehicle changes between 100° and −50° in the whole integrated navigation experiment, and it has a big range of change. The estimation values of the heading angle based on the three filtering methods can all track the change of the true heading angle, but the estimation accuracies are different.

It can be seen from Figure 8 that the estimation values of heading angle error of the federated Kalman filter are the biggest. The estimation values reach the maximum value of 9.38° around 100 s, and after that they reduce to 2.68° at 600 s. The estimation values of the adaptive federated Kalman filter are smaller than that of the federated Kalman filter. Its estimation values reach the maximum value of 6.31° around 100 s, and after that they reduce to 1.05° at 600 s. Comparatively, the estimation values of the adaptive federated IMM filter is the smallest. Its estimation values reach the negative maximum value of −0.63° around 50 s, then after that the estimation values tend to stabilize around 0.26°, and they are little influenced by the change of external disturbances. This is because the proposed adaptive federated IMM filter uses different models for each local system, when the external disturbances change the model for each local system can switch in time. Therefore, the adaptive federated IMM filter can use the most accurate model to describe the current state of each local system, and the estimation values of heading angle error can be effectively reduced.

**Figure 9:**
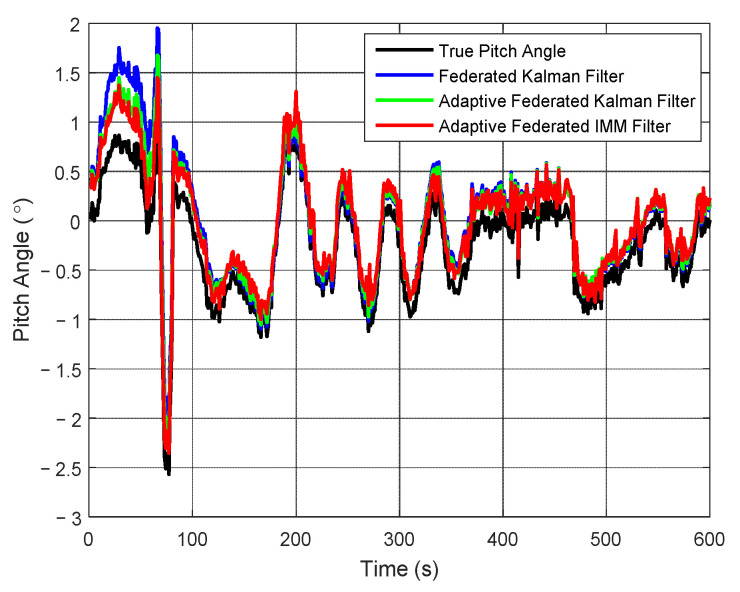
The estimation curves of pitch angle.

**Figure 10:**
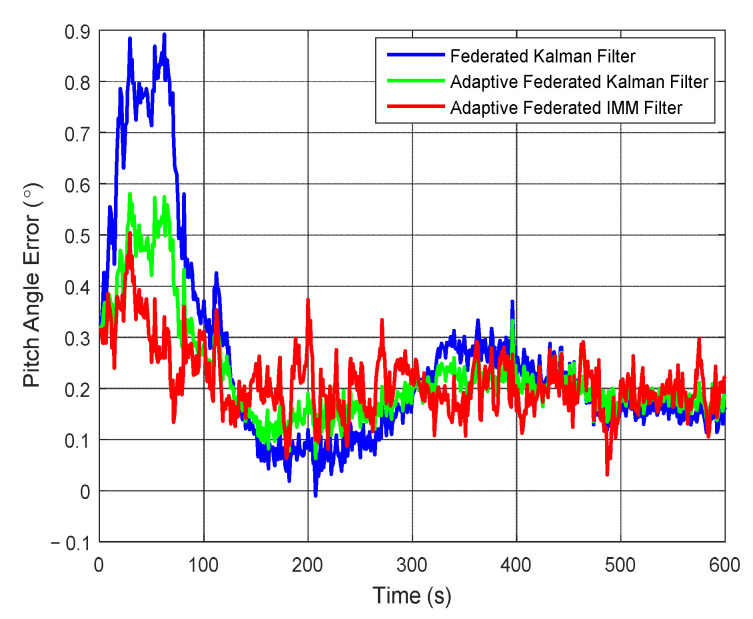
The estimation curves of pitch angle error.

The estimation curves of pitch angle and pitch angle error are presented in Figure 9 and Figure 10, respectively. In Figure 9, the pitch angle of the test vehicle changes between 2° and −3° in the integrated navigation experiment. Although the estimation values of pitch angle from the three filtering methods are close to the true pitch angle, there still are some differences.

It can be seen from Figure 10 that when using the federated Kalman filter, the estimation values of pitch angle error reach the maximum value of 0.89° around 60 s, and after that they begin to reduce to smaller values with some fluctuations. When using the adaptive federated Kalman filter, the estimation values reach the maximum value of 0.57° around 60 s, and after that they begin to reduce with fluctuations smaller than that of the federated Kalman filter. In contrast, when using the adaptive federated IMM filter, the estimation values reach the maximum value of 0.50° around 30 s, and after that they quickly form pattern more stable than the other two filtering methods.

**Figure 11:**
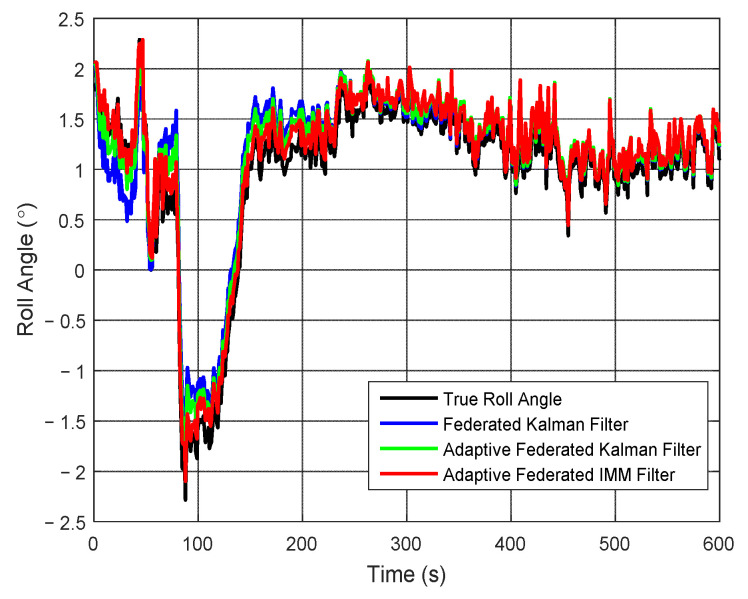
The estimation curves of roll angle.

**Figure 12:**
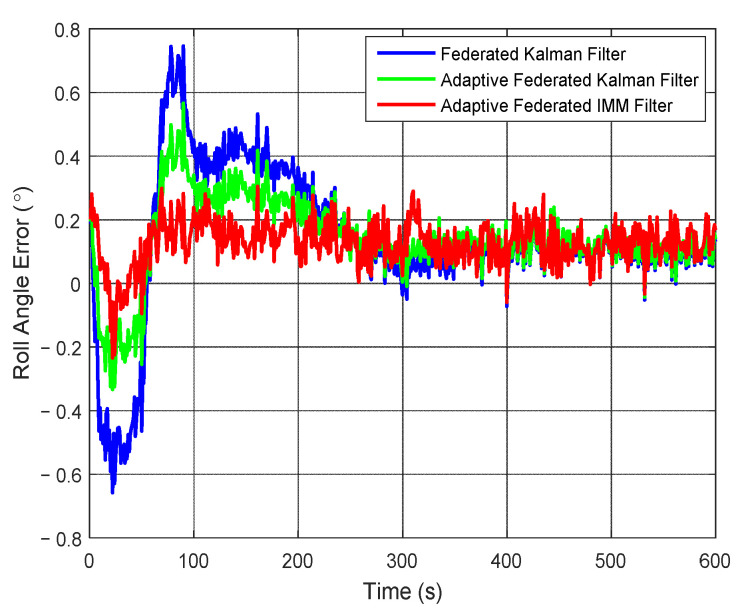
The estimation curves of roll angle error.

Correspondingly, the estimation curves of roll angle and roll angle error are shown in Figure 11 and Figure 12, respectively. As shown in Figure 11, the roll angle of the test vehicle changes between 2.5° and −2.5° in the integrated navigation experiment. The estimation values of the roll angle of all three filtering methods are close to the true roll angle, but there are still some differences.

It can be seen from Figure 12 that when using the federated Kalman filter, the estimation values of roll angle error reach a negative maximum value of −0.65° around 20 s and a positive maximum value of 0.75° around 90 s, and they are greatly affected by the external noises. When using the adaptive federated Kalman filter, the estimation values of roll angle error reach a negative maximum value −0.33° around 20 s and a positive maximum value of 0.57° around 90 s, and they are less affected by the external noises. In contrast, when using the adaptive federated IMM filter, the estimation values only reach a negative maximum value of −0.22° around 20 s, and after that, the estimation values become distinctly more stable than those from the other two filtering methods.

**Figure 13:**
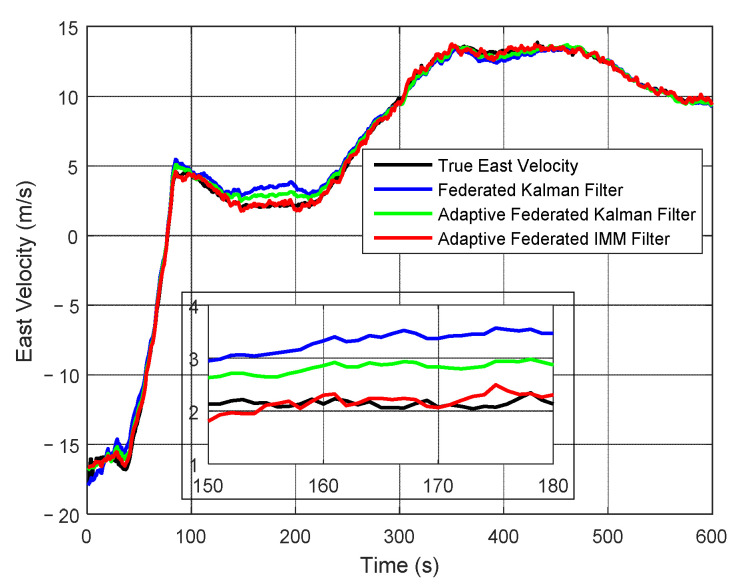
The estimation curves of east velocity.

**Figure 14:**
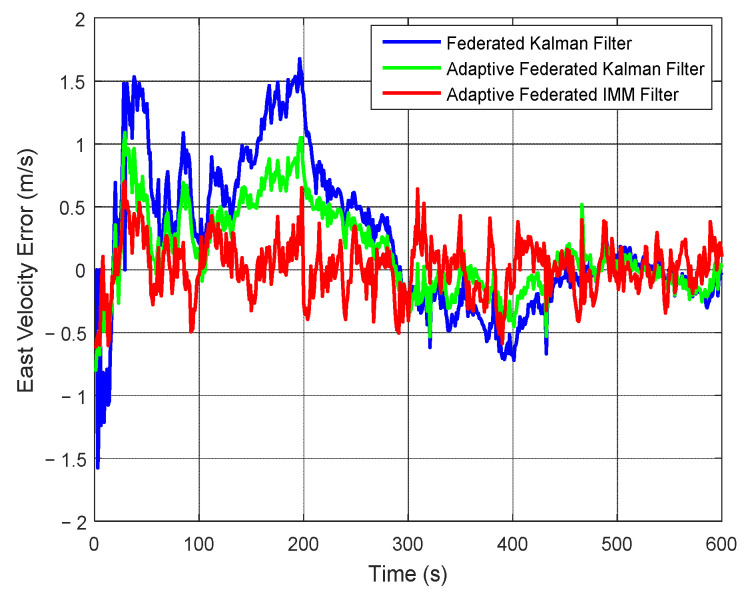
The estimation curves of east velocity error.

The estimation curves of east velocity and east velocity error are shown in Figure 13 and Figure 14, respectively. As shown in Figure 13, the east velocity changes between −20 m/s and 15 m/s. The estimation values of east velocity of three filtering methods can all track the change of the true east velocity, but their estimation accuracy is different. From the partially enlarged figure, the estimation values of east velocity of the adaptive federated IMM filter is the closest to the true east velocity. 

As shown in Figure 14, when using the federated Kalman filter, the estimation values of east velocity error have large fluctuations within 300 s, with a maximum value of 1.68 m/s around 200 s. When using the adaptive federated Kalman filter, the estimation values reach a maximum value of 1.05 m/s around 200 s, and the fluctuations are smaller than those of the federated Kalman filter. Comparatively, when using the adaptive federated IMM filter, the estimation values keep stable in the whole process, and its fluctuations are the smallest among the three filtering methods. This is because the proposed adaptive federated IMM filter can switch the model of each local system in time when the external disturbances change, and the most accurate model can be established to describe the current motion state.

**Figure 15:**
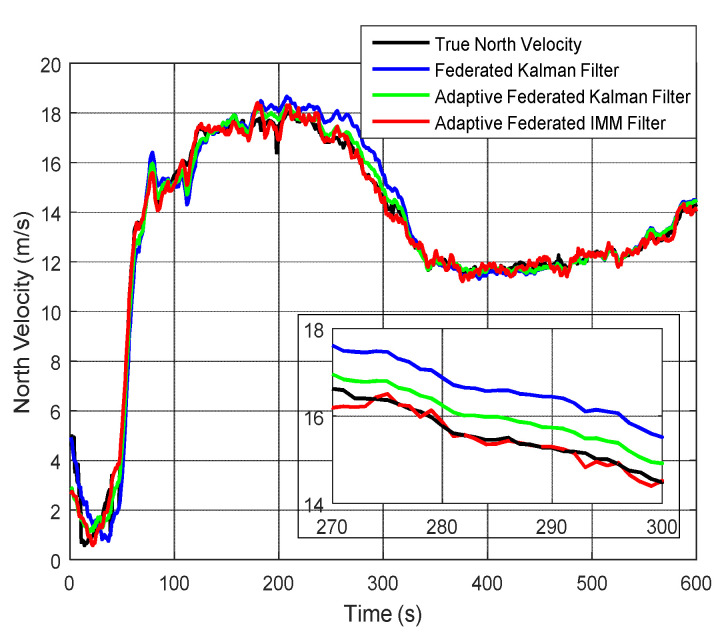
The estimation curves of north velocity.

**Figure 16:**
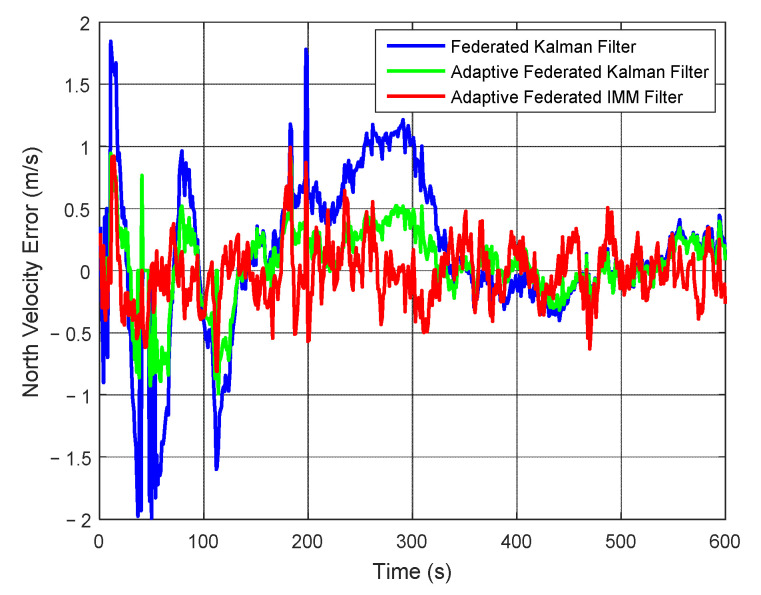
The estimation curves of north velocity error.

The estimation curves of north velocity and north velocity error are shown in Figure 15 and Figure 16, respectively. From Figure 15, the north velocity changes between 0 and 20 m/s. It can be seen from the partially enlarged figure that the estimation values of north velocity by using the adaptive federated IMM filter is the closest to the true north velocity compared with that by using the other two filtering methods.

As shown in Figure 16, when using the federated Kalman filter, the estimation values of north velocity error have a couple of big fluctuations within 350 s, and the estimation values reach a negative maximum value of −1.99 m/s around 50 s. Therefore, its estimation values are greatly affected by the external noises during the integrated navigation experiment. When using the adaptive federated Kalman filter, the estimation values reach a negative maximum value of −0.99 m/s around 110 s, and the fluctuations are distinctly smaller than those using the federated Kalman filter. Comparatively, when using the adaptive federated IMM filter, its estimation values have the smallest fluctuations among all three filtering methods.

**Figure 17:**
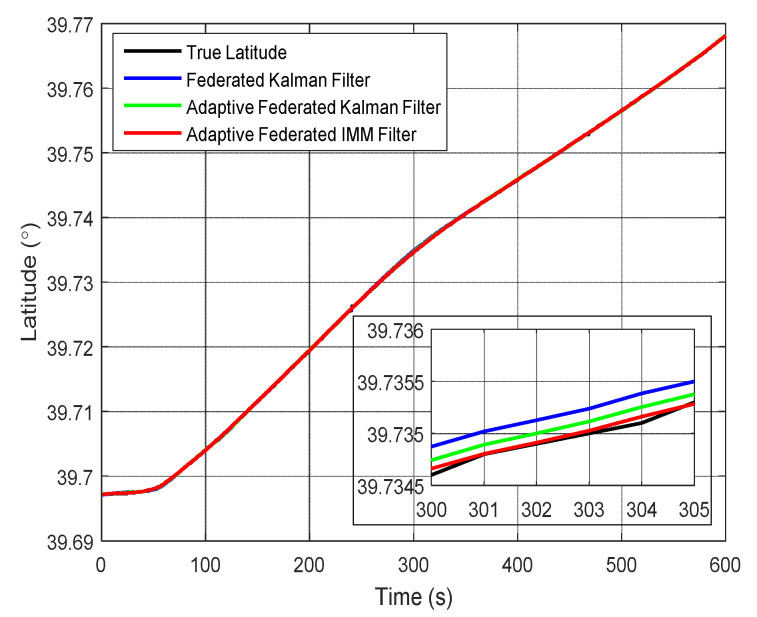
The estimation curves of latitude.

**Figure 18:**
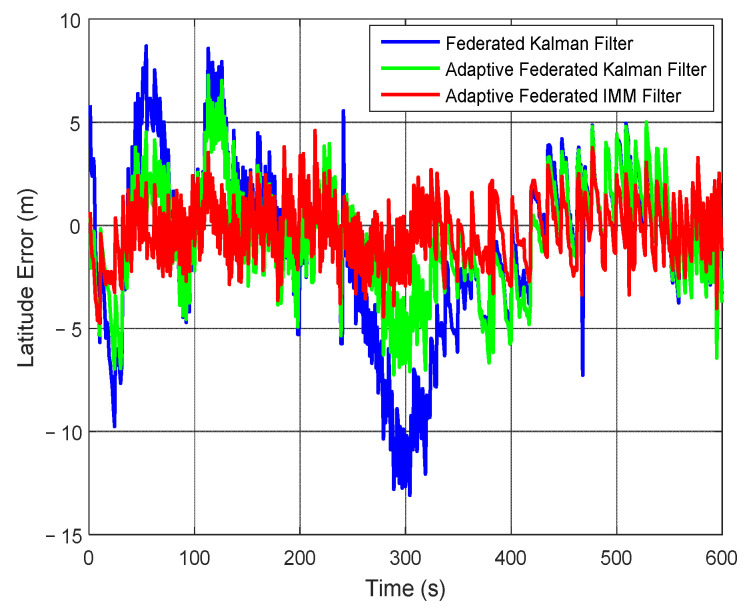
The estimation curves of latitude error.

The estimation curves of latitude and latitude error are shown in Figure 17 and Figure 18, respectively. Because the position is the integral of velocity, the estimation curves of position are smoother than that of velocity. From Figure 17, the estimation curves by using all the filtering methods can track the change of latitude, but the estimation values of latitude by using the adaptive federated IMM filter are the closest to the true latitude among the three filtering methods.

From Figure 18, the estimation values of latitude error by using the federated Kalman filter exist big fluctuations and they reach a negative maximum value of −13.10 m around 300 s. The estimation values by using the adaptive federated Kalman filter reach a maximum value of 7.29 m around 100 s, and its estimation values have smaller fluctuations than that using the federated Kalman filter. In contrast, when using the adaptive federated IMM filter, the estimation values of latitude error are distinctly more stable than that using the other two filtering methods.

**Figure 19:**
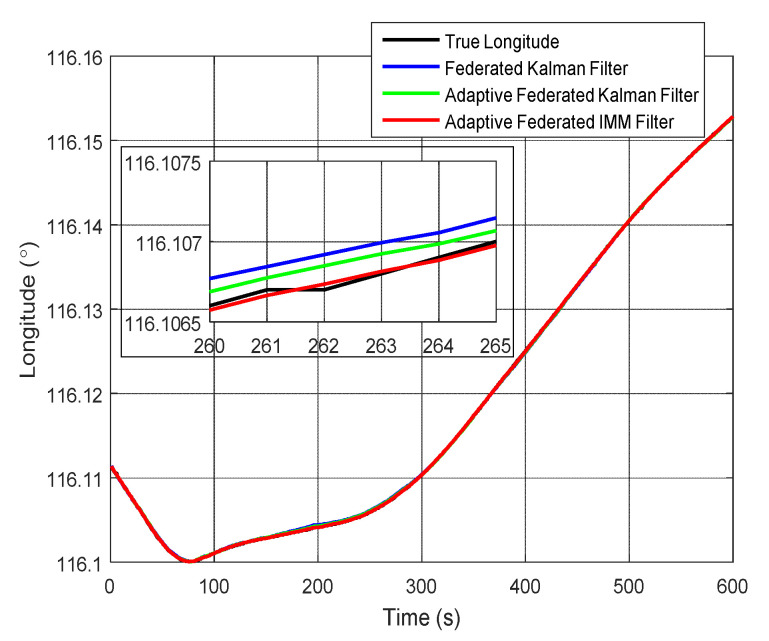
The estimation curves of longitude.

**Figure 20:**
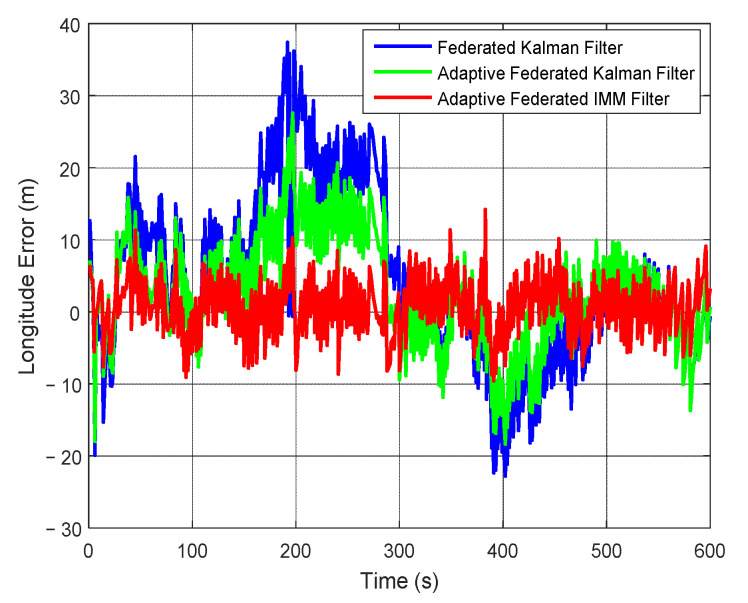
The estimation curves of longitude error.

Correspondingly, the estimation curves of longitude and longitude error are shown in Figure 19 and Figure 20, respectively. As shown in Figure 19, the estimation values of longitude by using the adaptive federated IMM filter are closer to the true longitude compared with that using the federated Kalman filter and the adaptive federated Kalman filter.

Moreover, it can be seen from Figure 20 that when using the federated Kalman filter the estimation values of longitude error reach a maximum value of 37.44 m around 200 s, and they are greatly affected by the external disturbances. When using the adaptive federated Kalman filter, the estimation values reach a maximum value of 27.59 m around 200 s, and they are less affected by the external disturbances than that using the federated Kalman filter. Comparatively, when using the adaptive federated IMM filter, the estimation values of longitude error are the least affected by the external disturbances and they keep stable in the whole process of integrated navigation.

**Figure 21:**
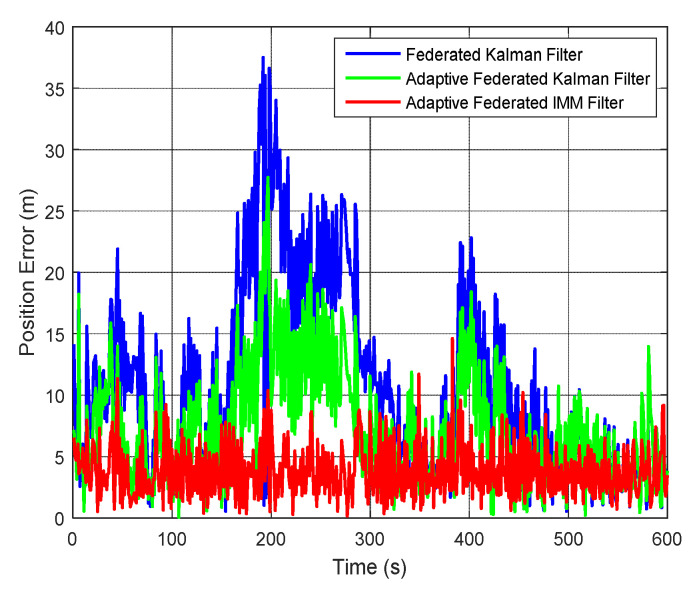
The estimation curves of position error.

Consequently, the estimation curves of position error are shown in Figure 21. It can be seen that the estimation curves of position error of the federated Kalman filter have some large fluctuations during the 600 s of integrated navigation, and its estimation values reach a maximum value of 37.49 m around 200 s. When using the adaptive federated Kalman filter, the estimation values reach a maximum value of 27.73 m around 200 s, and the fluctuations are smaller than that using the federated Kalman filter. By contrast, when using the adaptive federated IMM filter, the estimation values of position error are the least affected by the external disturbances, and the fluctuations are the smallest among the three filtering methods. The mean absolute errors (MAEs) of position error when using the federated Kalman filter, the adaptive federated Kalman filter, and the adaptive federated IMM filter are 10.87 m, 7.36 m, 3.82 m, respectively. As a result, the position error when using the adaptive federated IMM filter was reduced by 64.86% and 48.10%, respectively, compared with the federated Kalman filter and the adaptive federated Kalman filter. In summary, the MAEs and standard deviations (STDs) of integrated navigation errors when using the three filtering methods are listed in Table 2.

**Figure 22:**
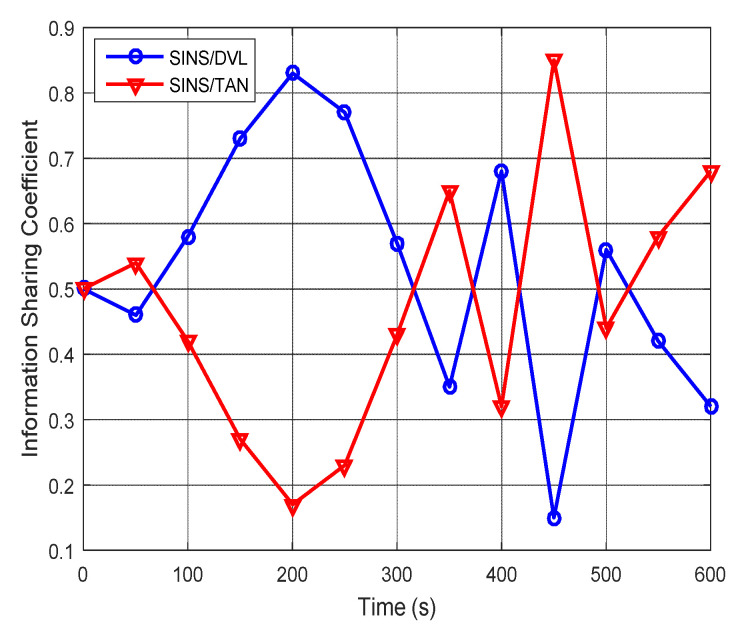
The values of the information sharing coefficient.

In the AUV integrated navigation experiment, the external environment is complex and time-varying, and the disturbances in the underwater environment are bigger than those on the land. Therefore, the outputs of the DVL and the TAN are easily disturbed by the underwater environment, and the output accuracy of the DVL and the TAN is decreased and is unstable. When the beams of the DVL are unable to reach the seabed, the accuracy of the SINS/DVL measurement model is decreased. When the underwater map information is inaccurate in some places, or the underwater terrain is relatively flat, the SINS/TAN measurement model is unable to provide accurate position information. To solve this problem, the proposed adaptive federated IMM filter is designed to adaptively adjust the information sharing coefficients of the local SINS/DVL system and the local SINS/TAN system during the integrated navigation experiment. Figure 22 shows the values of the information sharing coefficient in the adaptive federated IMM filter. It can be seen that, when the performance of each local system changes, the information sharing coefficients of the local SINS/DVL system and the local SINS/TAN system are adjusting in real time. 

**Figure 23:**
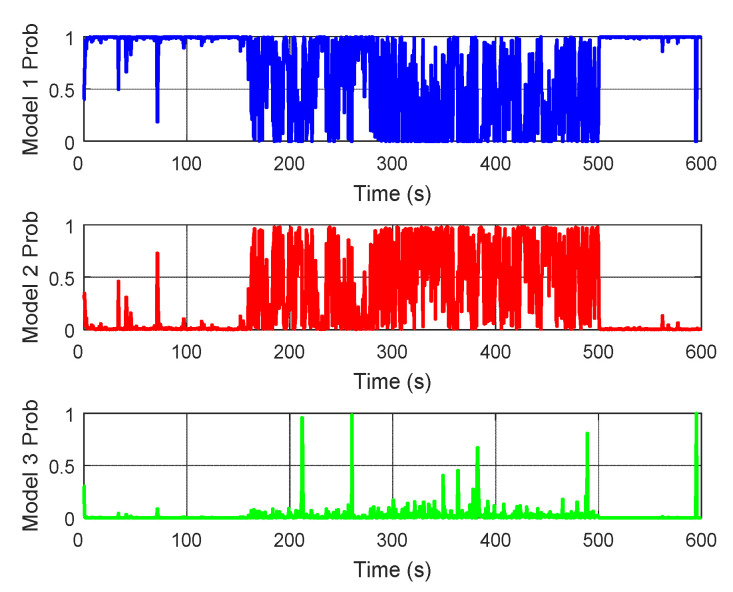
The model probability of the local SINS/DVL system.

**Figure 24:**
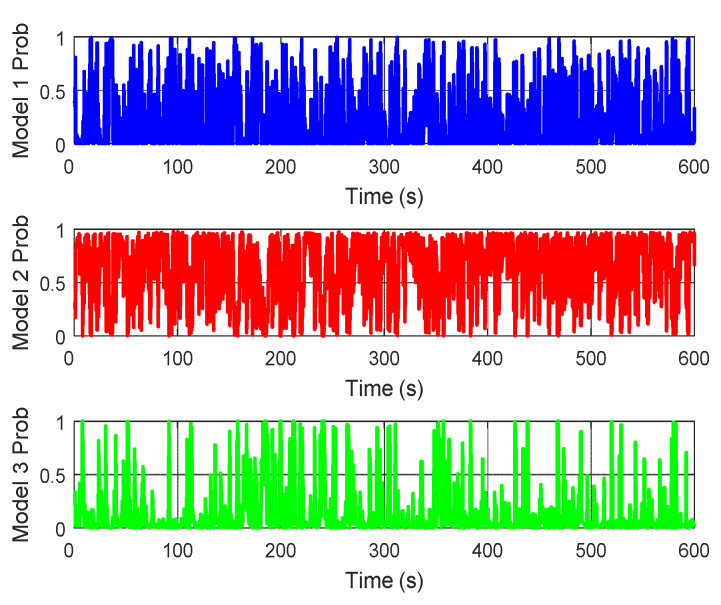
The model probability of the local SINS/TAN system.

Moreover, the model probabilities of the local SINS/DVL system and the local SINS/TAN system in the proposed adaptive federated IMM filter are presented in Figure 23 and Figure 24, respectively. It can be seen that during the process of integrated navigation, the model probability of each local system switches in real-time, and the most accurate model can be established to describe the current state of the local system.

The three filtering methods are coded with C++ and the integrated navigation experiments are run on a computer with Intel Core i7-6500 CPU at 2.50 GHz. The implementation times of the three filtering methods in single step run (a single prediction and update step) are listed in Table 3. It can be seen from Table 3 that the implementation time of the proposed adaptive federated IMM filter in single step run is 2.96 × 10^−3^ s. Therefore, the proposed filtering method can ensure running in real-time during the integrated navigation experiments, and it is fast enough in real-world applications. 

**Figure 25:**
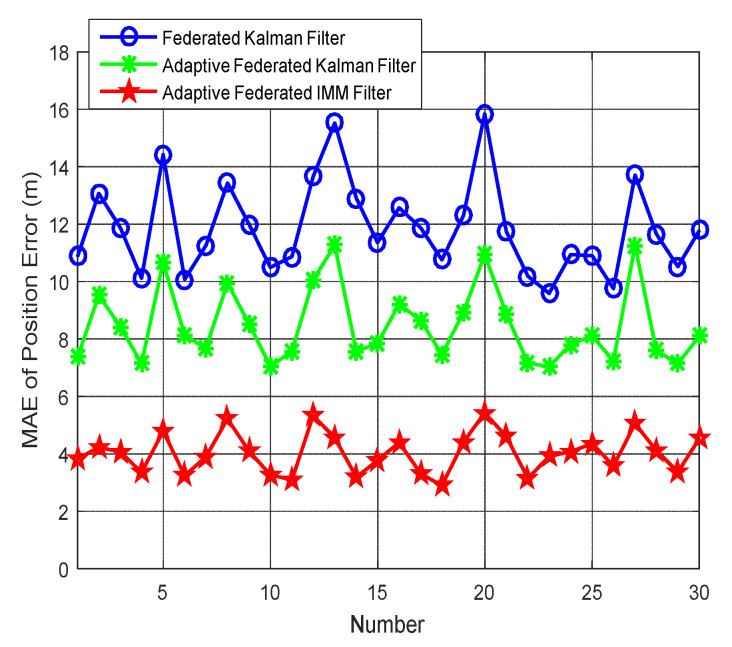
The mean absolute errors (MAEs) of position errors in 30 groups of integrated navigation experiments.

In order to further compare the federated Kalman filter, the adaptive federated Kalman filter, and the adaptive federated IMM filter in the process of integrated navigation, this study designed a total of 30 groups of vehicle integrated navigation experiments on a real platform. The test vehicle’s moving trajectories, running speeds, and conditions of the road surface are entirely different in those experiments. In each experiment, the SINS, GNSS receiver, odometer were restarted before working. The line charts of the MAEs of position errors in 30 groups of integrated navigation experiments are shown in Figure 25, and the MAEs of position errors in 30 groups of integrated navigation experiments are listed in Table 4.

From Figure 25 and Table 4, it can be seen that in those vehicle integrated navigation experiments, the position errors determined when using the adaptive federated IMM filter are obviously smaller than those determined when using the federated Kalman filter and the adaptive federated Kalman filter. By calculation, the mean position errors of 30 groups of integrated navigation experiments by using the federated Kalman filter, the adaptive federated Kalman filter, and the adaptive federated IMM filter are 11.86 m, 8.47 m, and 4.03 m, respectively. As a result, the mean position error determined when using the adaptive federated IMM filter was reduced by 66.02% compared with the federated Kalman filter and 52.42% compared with the adaptive federated Kalman filter. The position errors of those vehicle integrated navigation experiments further illustrate that the proposed adaptive federated IMM filter can effectively improve the accuracy of integrated navigation, and it has obvious advantages compared with the federated Kalman filter and the adaptive federated Kalman filter.

## 6. Conclusions

In this paper, an adaptive federated IMM filter for the AUV integrated navigation system is presented. The adaptive federated IMM filter combines an adaptive federated filter and IMM algorithm to improve the accuracy and reliability of the AUV integrated navigation system in the complex underwater environment. The information sharing coefficient of the adaptive federated IMM filter is adaptively adjusted when the performance of each local system changes. Meanwhile, each local system of the integrated navigation system includes different models, with the change of the underwater environment, and the adaptive federated IMM filter can use the most accurate mixed model to describe the current state of the local system. Furthermore, an AUV integrated navigation system model that includes the system error dynamics model, SINS/DVL and SINS/TAN measurement models was established. 

In order to verify the effectiveness of the adaptive federated IMM filter, a total of 30 groups of vehicle integrated navigation experiments on a real platform were performed. The experimental results show that the proposed adaptive federated IMM filter has obvious advantages compared with the federated Kalman filter and the adaptive federated Kalman filter. The research presented in this paper provides a new idea for AUV integrated navigation system in the underwater environment. Further work will focus on the practical application of AUV in the underwater environment to validate the performance of the proposed method.

## Figures and Tables

**Table 1:** Specifications of the instruments in the integrated navigation experiment.

Instruments	Parameters	Accuracy
SINS	three-axis gyro random constant drifts three-axis gyro random noise three-axis accelerometer random constant biases three-axis accelerometer random noise	1.0°/h (1σ) 0.25°/h^1/2^ (1σ) 0.1 mg (1σ) 0.04 μg /Hz^1/2^ (1σ)
Odometer	Velocity	120 pulse/circle
GNSS receiver	Position	10 m (1σ)

**Table 2:** The mean absolute errors (MAEs) and standard deviations (STDs) of integrated navigation errors by the three filtering methods.

Parameter Errors	Federated Kalman Filter		Adaptive Federated Kalman Filter		Adaptive Federated IMM Filter	
	MAE	STD	MAE	STD	MAE	STD
Heading Angle (°)	3.99	1.97	1.54	1.35	0.33	0.12
Pitch Angle (°)	0.25	0.20	0.22	0.10	0.21	0.07
Roll Angle (°)	0.14	0.23	0.14	0.13	0.13	0.07
East Velocity (m/s)	0.23	0.59	0.14	0.34	0.02	0.22
North Velocity (m/s)	0.14	0.60	0.05	0.30	−0.02	0.25
Latitude (m)	−0.99	4.20	−0.62	2.79	−0.26	1.64
Longitude (m)	4.58	11.68	2.76	7.79	0.78	3.97
Position (m)	10.87	7.59	7.36	4.72	3.82	2.13

**Table 3:** The implementation times of the three filtering methods in single step run.

Filtering Methods	Time (s)
Federated Kalman Filter	9.61 × 10^−4^
Adaptive Federated Kalman Filter	9.72 × 10^−4^
Adaptive Federated IMM Filter	2.96 × 10^−3^

**Table 4:** The MAEs of position errors (m) in 30 groups of integrated navigation experiments.

Number	Federated Kalman Filter (m)	Adaptive Federated Kalman Filter (m)	Adaptive Federated IMM Filter (m)
1	10.87	7.36	3.82
2	13.06	9.51	4.23
3	11.87	8.43	4.06
4	10.12	7.14	3.35
5	14.42	10.67	4.78
6	10.07	8.11	3.24
7	11.25	7.68	3.89
8	13.43	9.95	5.21
9	11.98	8.51	4.09
10	10.49	7.04	3.26
11	10.86	7.56	3.08
12	13.65	10.04	5.33
13	15.51	11.27	4.56
14	12.90	7.57	3.18
15	11.35	7.84	3.77
16	12.57	9.21	4.39
17	11.87	8.64	3.31
18	10.76	7.45	2.89
19	12.30	8.93	4.41
20	15.83	10.94	5.42
21	11.74	8.87	4.60
22	10.18	7.16	3.14
23	9.59	7.03	3.91
24	10.94	7.81	4.07
25	10.89	8.10	4.35
26	9.75	7.23	3.57
27	13.70	11.25	5.04
28	11.66	7.59	4.11
29	10.52	7.14	3.36
30	11.78	8.10	4.53
